# cDNA Library Screening Identifies Protein Interactors Potentially Involved in Non-Telomeric Roles of *Arabidopsis* Telomerase

**DOI:** 10.3389/fpls.2015.00985

**Published:** 2015-11-12

**Authors:** Ladislav Dokládal, David Honys, Rajiv Rana, Lan-Ying Lee, Stanton B. Gelvin, Eva Sýkorová

**Affiliations:** ^1^Mendel Centre for Plant Genomics and Proteomics, Central European Institute of Technology and Faculty of Science, Masaryk UniversityBrno, Czech Republic; ^2^Institute of Biophysics – Academy of Sciences of the Czech Republic v.v.i.Brno, Czech Republic; ^3^Institute of Experimental Botany – Academy of Sciences of the Czech Republic v.v.i.Prague, Czech Republic; ^4^Department of Biological Sciences, Purdue University, West LafayetteIN, USA

**Keywords:** telomerase, nuclear poly(A)-binding protein, telobox, metallothionein 2A, MODIFIER OF snc1, putative nuclear DNA-binding protein G2p, oxidation-related zinc finger 2 protein, BiFC

## Abstract

Telomerase-reverse transcriptase (TERT) plays an essential catalytic role in maintaining telomeres. However, in animal systems telomerase plays additional non-telomeric functional roles. We previously screened an *Arabidopsis* cDNA library for proteins that interact with the C-terminal extension (CTE) TERT domain and identified a nuclear-localized protein that contains an RNA recognition motif (RRM). This RRM-protein forms homodimers in both plants and yeast. Mutation of the gene encoding the RRM-protein had no detectable effect on plant growth and development, nor did it affect telomerase activity or telomere length *in vivo*, suggesting a non-telomeric role for TERT/RRM-protein complexes. The gene encoding the RRM-protein is highly expressed in leaf and reproductive tissues. We further screened an *Arabidopsis* cDNA library for proteins that interact with the RRM-protein and identified five interactors. These proteins are involved in numerous non-telomere-associated cellular activities. In plants, the RRM-protein, both alone and in a complex with its interactors, localizes to nuclear speckles. Transcriptional analyses in wild-type and *rrm* mutant plants, as well as transcriptional co-analyses, suggest that TERT, the RRM-protein, and the RRM-protein interactors may play important roles in non-telomeric cellular functions.

## Introduction

Telomeres are nucleoprotein structures at the ends of eukaryotic chromosomes, distinguishing these ends from double strand DNA breaks (DDBs) and protecting them from the DNA damage repair (DDR) machinery. Due to the “end replication problem” (Olovnikov, 1971; Watson, 1972; Olovnikov, 1973) telomeres are shortened in each round of replication until they are too short to function, leading to cell senescence (Levy et al., 1992) and apoptosis (Harley et al., 1990; Counter et al., 1992). Thus, telomeres limit cellular proliferative capacity and act as a biological “clock.” On the other hand, in cells with high proliferative need such as animal embryonic, stem, and cancer cells (reviewed in Blasco, 2005), or plant meristemic cells (Fitzgerald et al., 1996), telomere shortening is compensated by the action of telomerase, a conserved ribonucleoprotein complex with a reverse transcriptase subunit (Greider and Blackburn, 1985, 1987). Telomerase consists of two core subunits, telomerase RNA (TR) and telomerase reverse transcriptase (TERT), that are associated with several additional proteins not crucial for enzymatic activity. The TERT subunit has an evolutionary conserved primary structure which in most organisms can be further divided into N-terminal domains TEN (telomerase essential N-terminal) and TRBD (TR binding domain), a central reverse transcriptase (RT) domain, and a C-terminal extension (CTE; reviewed in Sykorova and Fajkus, 2009).

The fact that telomerase influences cellular life span and plays a role in various types of cancer intensified research in this field. Surprisingly, telomerase in mammalian cells influences tumourigenesis by additional mechanisms independent of telomere synthesis (reviewed in Majerska et al., 2011). These so-called non-telomeric functions of telomerase regulate processes such as apoptosis, cellular proliferation, and cell cycle regulation, generally by altering gene expression, or DDR by *de novo* telomere addition to the sites of DDB.

For the above reasons it is of great interest to study mechanisms and interactions through which telomerase is regulated, and by which telomerase regulates cellular functions other than telomere synthesis.

Telomerase from *Arabidopsis thaliana* represents a suitable model, especially because of the availability of viable T-DNA insertion mutants that are typically exploited in these types of studies. Classically, changes in telomere length and telomerase activity are measured in a particular mutant, which may lead to direct identification of important telomerase regulators. However, this approach may not detect interactors crucial for mediating non-telomeric activities of telomerase. For this purpose, methods such as tandem affinity purification or cDNA library screening may be more suitable.

The N- and C-terminal portions of TERT represent potential interacting targets for telomerase regulatory proteins. The CTE is highly conserved among vertebrates and plants and contains regions important for intracellular trafficking of human TERT, including a nuclear export signal, 14-3-3, and CRM1 binding sites (Seimiya et al., 2000). In our previous work we screened for *Arabidopsis* CTE protein–protein interactions against a cYFP-tagged *Arabidopsis* cDNA library in tobacco BY-2 protoplasts and identified two interacting partners, an armadillo/β-catenin-like repeat containing protein (encoded by At4g33945) interacting with CTE in the cytoplasm, and an RRM-containing protein (encoded by At5g10350; RRM) that interacts with the CTE in nuclei (Lee et al., 2012).

How telomerase executes its non-canonical activities and on which levels it regulates expression of its target genes are poorly understood. One possibility is regulation on the level of mRNA. The RRM protein belongs to a subfamily of *Arabidopsis* nuclear poly(A) binding proteins; that are characterized by a single RRM domain close to the C-terminus (reviewed in Eliseeva et al., 2013). The human nuclear poly(A) binding protein PABPN1 is implicated in a variety of mRNA stabilization and degradation processes, such as stimulation of poly(A) synthesis by poly(A) polymerase, protection of growing poly(A) chains from degradation, defining the length of growing poly(A) chains, and mRNA export (Wahle and Ruegsegger, 1999; Keller et al., 2000; Kuhn et al., 2009). In addition to RNA binding, the RRM domain may be responsible for interactions with other proteins or DNA (reviewed in Krietsch et al., 2013). These observations support the hypothesis that the interaction between TERT and RRM might be a mechanism by which telomerase could affect many cellular processes.

Here, we present further characterization of the RRM protein and discuss its potential physiological role in telomerase involvement in non-telomeric activities. We describe the interaction profile of the RRM protein and analyze telomere length, telomerase activity, and changes in gene expression in T-DNA insertion mutants that disrupt the *RRM* gene.

## Materials and Methods

### Plant Material

*Arabidopsis* T-DNA insertion lines SALK_096285 (*rrm-1*) and SALK_116646C (*rrm-2*) were obtained from the Nottingham *Arabidopsis* Stock Centre. Both mutant and wild-type (Col-0) *A. thaliana* seeds were surface sterilized and germinated on 0.8% (w/v) agar plates supplemented with 1/2 Murashige and Skoog medium (MS; cat. n. M0255.0050; Duchefa ^1^) and 1% (w/v) sucrose. Seedlings were potted after 7 days and further grown in the conditions of 16 h light, 21°C and 8 h dark, 19°C, illumination 150 μmol m^-2^ s^-1^. Individual plants from each T-DNA insertion line were genotyped (see Supplementary Table S1 for primer sequences) and after selection of homozygous mutant plants, three subsequent generations were grown.

### Telomere Length and Telomerase Activity Analyses

The terminal restriction fragment (TRF) analysis using Southern blot hybridization, the conventional TRAP (telomere-repeat-amplification-protocol) and the quantitative TRAP assays were performed as described (Fojtova et al., 2011). Mean telomere length values were calculated using TeloTool software (Gohring et al., 2014).

### Entry Clone Generation

Sequences encoding full-length RRM (At5g10350) and G2p (At3g51800) proteins were amplified from 7-days-old seedling cDNA by Phusion HF DNA polymerase (Finnzymes^2^) according to the manufacturer’s instructions. Sequences encoding RRM fragments [RRM-1(1–81); RRM-2(1–169); RRM-3(170–217); RRM-4(82–217); RRM-5(82–169)] were sub-cloned using KAPA Taq DNA polymerase (Kapabiosystems^3^) and a pGADT7-DEST::RRM construct as a template. Primers used for cloning are listed in Supplementary Table S1. PCR products were precipitated using PEG and cloned into pDONR/Zeo (Invitrogen^4^). The MT2A (At3g09390) coding sequence was sub-cloned into pDONR/zeo from the cYFP cDNA library clone 212M1 (Lee et al., 2012). Entry clones encoding HSP70-1 (stock no. GC104920, At5g02500) and OZF2 (stock no. G10332, At4g29190; Yamada et al., 2003) were obtained from the ABRC^5^. Entry clones encoding AtTERT (At5g16850) fragments TEN(1–233), RID1(1–271), Fw3N-NLS(229–582), RT(597–987), and CTE2(958–1123) were prepared previously (Zachova et al., 2013).

### Yeast Two Hybrid Analysis

Yeast two-hybrid experiments were performed using the Matchmaker^TM^ GAL4-based two-hybrid system (Clontech^6^). cDNA sequences encoding RRM protein (full-length and fragments), TERT fragments, G2p, MT2A, HSP70-1, and OZF2 were subcloned from their entry clones into the destination vectors pGADT7-DEST and pGBKT7-DEST. Each bait/prey combination was co-transformed into *Saccharomyces cerevisiae* PJ69-4a and yeast two hybrid analysis was performed as described in Schrumpfová et al. (2014). Protein expression was verified by immunoblotting using mouse anti-HA (kindly provided by Dr. Vojtěšek) or mouse anti-myc primary antibodies and HRP-conjugated anti-mouse secondary antibody (both Sigma-Aldrich^7^).

### Bimolecular Fluorescence Complementation and Screening of cYFP cDNA Library

The constructs nYFP-TERT(CTE2), n/cYFP-TERT(RID1), and cYFP-RRM were created previously (Lee et al., 2012; Schrumpfová et al., 2014). The RRM, G2p, HSP70-1, and OZF2 coding sequences were subcloned from their entry clones into the destination vector pSAT4-DEST-nEYFP-C1 (Gelvin laboratory stock number pE3136). To visualize RRM subcellular localization, the RRM coding sequence was subcloned into the destination vector p2YGW7, generating a YFP tag at the N-terminus of the protein. The nYFP-RRM construct was screened against a cYFP cDNA library for protein–protein interactions in tobacco BY-2 protoplasts as described (Lee et al., 2012).

Tobacco BY-2 protoplasts were isolated and transfected as previously described (Tenea et al., 2009; Lee et al., 2012). *Arabidopsis* leaf protoplasts were isolated and transfected as described by Wu et al. (2009). To label cell nuclei, we co-transfected a plasmid expressing mRFP fused to the nuclear localization signal of the VirD2 protein from *Agrobacterium tumefaciens* (mRFP-VirD2NLS; Citovsky et al., 2006). To label nuclear speckles, a pSRp30-RFP nuclear speckles marker (Lorkovic et al., 2004) was co-transfected. Transfected protoplasts were incubated in the dark at room temperature overnight, and observed for fluorescence using a Zeiss AxioImager Z1 epifluorescence microscope (Tobacco BY-2) or a Leica SPE confocal scanning light microscope (*Arabidopsis*). As a negative control, we used the constructs nYFP- and cYFP-GAUT10 (At2g20810). Protein expression was tested by immunoblotting using mouse anti-GFP primary antibody (Roche^8^) and HRP-conjugated anti-mouse secondary antibody (Sigma-Aldrich). Proteins were extracted from protoplasts into an extraction buffer (50 mM Na_2_HPO_4_, 10 mM EDTA, 0.1% Triton X-100, 10 mM 2-Mercaptoethanol, 1x Proteinase inhibitors cocktail, 1 mM PMSF) by vortexing.

### RNA Isolation and RT-qPCR Analysis

RNA from various *Arabidopsis* pollen developmental stages (Honys and Twell, 2004) was isolated using a Plant RNeasy Kit (Qiagen^9^) according to the manufacturer’s instructions, and further purified by DNaseI treatment (TURBO DNA-free kit, Thermo Fisher Scientific^10^). RNA isolation from other tissues of mutant or wild-type plants and reverse transcription were performed as described (Fojtova et al., 2011; Ogrocka et al., 2012). Calli were derived from 7-days-old seedlings, propagated on cultivation medium with 1 μg ml^-1^ 1-naphthaleneacetic acid and 1 μg ml^-1^ 2,4-dichlorophenoxyacetic acid, and subcultured monthly onto fresh medium. Transcript levels relative to a ubiquitin reference gene were analyzed using FastStart SYBR Green Master (Roche) and a 7300 Real-Time PCR System (Applied Biosystems^11^). A 1 μl aliquot of cDNA was added to the 20 μl reaction mix; the final concentration of each forward and reverse primer (Supplementary Table S1) was 0.5 μM. Reactions were performed in triplicate; PCR cycle conditions consisted of 10 min of initial denaturation followed by 40 cycles of 20 s at 95°C, 30 s at 55°C, and 1 min at 72°C. SYBR Green I fluorescence was monitored after each extension step. The amount of the respective transcript was determined for at least two biological replicates using the ΔΔCt method (Pfaﬄ, 2004).

### Identification of Genes Co-regulated with *RRM*

GENEVESTIGATOR (Nebion AG^12^) application (Hruz et al., 2008) was used to identify *in silico* genes co-regulated with *RRM* and genes encoding its interacting partners TERT, G2P, MOS1, OZF2, HSP70-1, and MT2A. Using this tool, we first defined conditions under which any of these genes shows an at least twofold change in transcript levels. We then searched for genes responding either in a similar (score 0 to 1) or opposite (score -1 to 0) manner on the same subset of defined conditions. Genes with a co-regulation level score either higher than 0.5 or lower than -0.5 were considered for further analyses. Telobox motifs in candidate genes were identified in the literature or by manually searching for the motifs AAACCCT, AACCCTA and their corresponding reverse complements, in the genomic region 1000 bp upstream of the translation start (ATG) site using publically available data at NCBI^13^ and/or the gene datasets from Wang et al. (2011). Putative protein–protein-interaction networks were visualized using STRINGv10^14^ (Szklarczyk et al., 2015).

## Results

### Verification of *In vivo* Interaction between the RRM Protein and the CTE Domain of AtTERT

**FIGURE 1:**
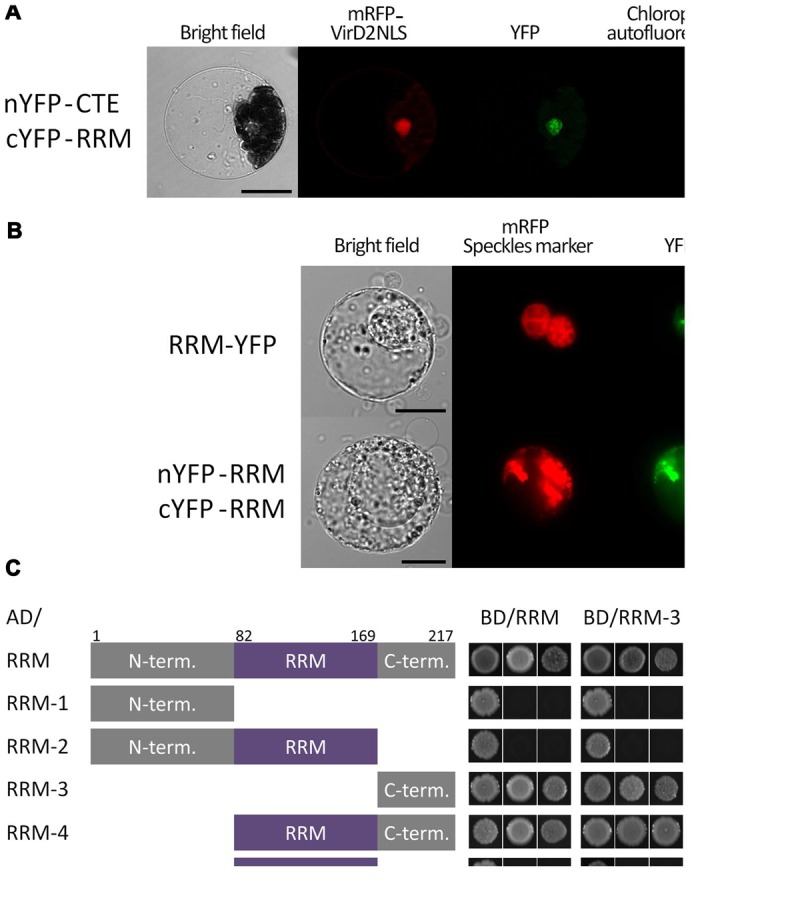
**Subcellular localization and dimerization of the RRM protein and verification of its interaction with the CTE domain of AtTERT. (A)** BiFC in *Arabidopsis* leaf protoplasts confirmed a nuclear interaction of the RRM protein with AtTERT CTE domain. Protoplasts were co-transfected with plasmids encoding nYFP-tagged TERT(CTE2), cYFP-tagged RRM, and mRFP-VirD2(NLS) to label cell nuclei; nYFP- and cYFP-GAUT10 constructs served as negative control (not shown). YFP fluorescence is shown in green, mRFP fluorescence in red, and chlorophyll autofluorescence in blue pseudocolor. Scale bar indicates 10 μm. **(B)** RRM-YFP co-localizes with a pSRp30-RFP nuclear speckle marker in tobacco BY-2 protoplasts. The same localization pattern was observed for interaction of nYFP-RRM with cYFP-RRM. YFP fluorescence is shown in green, and mRFP fluorescence of the VirD2(NLS) marker is shown in red. Scale bars indicate 20 μm. **(C)** A Y2H system was used to assess RRM dimerization. Two sets of plasmids carrying full-length RRM or indicated RRM segments fused to either the GAL4 DNA-binding domain (BD) or the GAL4 activation domain (AD) were constructed and introduced into *Saccharomyces cerevisiae* PJ69-4a carrying *His3* and *Ade2* reporter genes. Co-transformation with an empty vector served as a negative control (not shown). Full-length RRM protein self-interacted on both histidine and stringent adenine selection plates. The same result was observed for interactions of the RRM-4(82–217) and the RRM-3(170–217) fragments with the full-length RRM protein, each other, and themselves, suggesting that the RRM-protein C-terminus is responsible for protein homodimerization. None of the other fragments [RRM-1(1–81), RRM-2(1–169), and RRM-5(82–169)] showed interaction, although their successful expression was confirmed by immunoblotting (Supplementary Figure S1).

The RRM protein was identified as a putative interaction partner of AtTERT by screening a cYFP-tagged cDNA library using BiFC in tobacco BY-2 protoplasts (Lee et al., 2012). To test if this interaction is independent of the plant system used, we expressed both tagged partners in *Arabidopsis* leaf protoplasts and observed a positive BiFC signal in the nucleus (**Figure 1A**). We further employed the yeast-two-hybrid (Y2H) system, but no interaction was observed in yeast despite the expression of both proteins (Supplementary Figure S1). These results suggested that the *in vivo* interaction between the RRM and the AtTERT CTE domain was mediated by an additional plant protein or protein modification absent in yeast cells but present in telomerase-positive (BY-2) and telomerase-negative (*Arabidopsis* leaf) cells.

### The C-terminus of the RRM Protein is Responsible for RRM Dimerization

We tested the ability of the At5g10350 protein containing a single RRM domain to form homodimers using Y2H and BiFC analyses (**Figures 1B,C**). RRM dimerization was observed using BiFC in tobacco BY-2 protoplasts, where the interaction provided a pattern similar to that of full length RRM-YFP fusion protein that co-localized with the pSRp30-RFP nuclear speckles marker (**Figure 1B**). We further tested this interaction using Y2H where the RRM protein showed strong self-interaction using both histidine and stringent adenine growth selection (**Figure 1C**). To determine which part of the RRM molecule is responsible for dimerization, we prepared five constructs corresponding to various structural domains of the RRM protein. We tested them for interaction with full length RRM and with each other (**Figure 1C**) by Y2H analysis. The RRM-4(82–217) fragment comprising the RRM domain and the C-terminus, and the RRM-3(170–217) fragment with the C-terminus only, interacted with the full length RRM protein, each other, and themselves using both histidine and stringent adenine growth selection, suggesting that the C-terminus is responsible for protein homodimerization. The fragments RRM-1(1–81) – N-terminus, RRM-2(1–169) – N-terminus and the RRM domain, and RRM-5(82–169) – the RRM domain did not show positive Y2H signals, although their successful expression was confirmed by immunoblotting (Supplementary Figure S1).

### BiFC Screening of an *Arabidopsis* cDNA Library Identified Proteins that Interact with the RRM Protein

**FIGURE 2:**
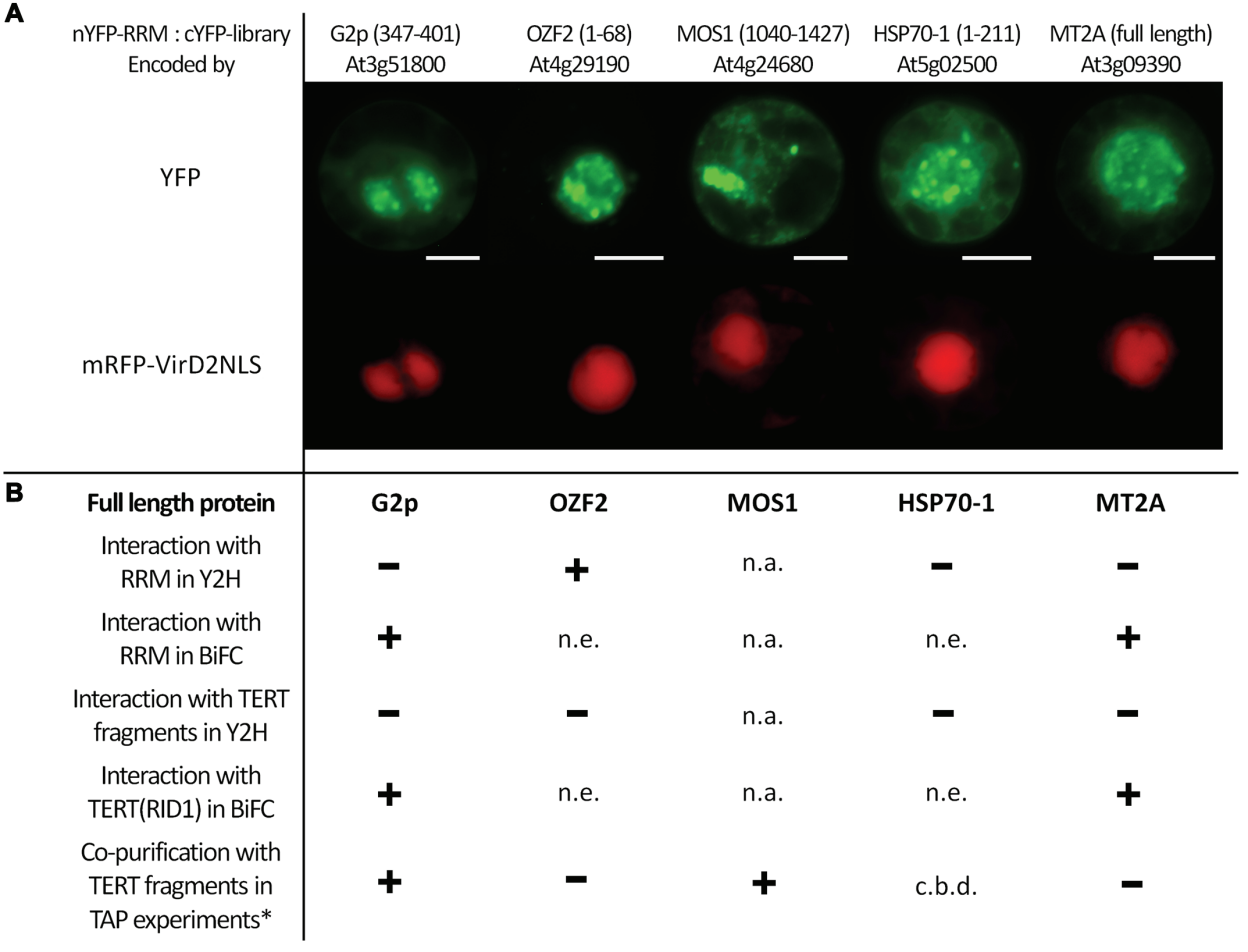
**BiFC screening of an *Arabidopsis* cDNA library identified interaction partners of the RRM protein. (A)** Interactions of nYFP-RRM with cYFP-tagged protein fragments of G2p(347–401), OZF2(1–68), MOS1(1040–1427), HSP70-1(1–211) and full-length MT2A protein identified by screening a cYFP-tagged cDNA library. Tobacco BY-2 protoplasts were co-transfected with plasmids encoding a mRFP-VirD2NLS nuclear marker, nYFP-RRM, and one of the five interacting cYFP-tagged proteins. YFP fluorescence is shown in green, and mRFP fluorescence is shown in red. Scale bars indicate 20 μm. **(B)** Summary of investigated protein–protein interactions of full-length G2p, OZF2, HSP70-1, and MT2A proteins with full-length RRM protein and TERT fragments. One of the fragments (RID1) was used in BiFC; all other TERT fragments were investigated using a Y2H system. Using the GAL4-based Y2H system in *S. cerevisiae* PJ69-4a carrying *His3* and *Ade2* reporter genes, we confirmed interaction only between OZF2-AD and RRM-BD on both histidine and stringent adenine selection plates. Other investigated combinations were negative, excluding the OZF2-BD construct that showed false positive interactions, and the HSP70-1-AD construct that was not expressed. Protein expression was checked by immunoblotting (Supplementary Figure S1). In addition to interaction of MT2A with full-length RRM protein shown on **(A)**, BiFC analysis in tobacco BY-2 protoplasts revealed positive interactions of MT2A with the TERT(RID1) fragment and also of full-length G2p protein with both full-length RRM protein, and the TERT(RID1) fragment (Supplementary Figure S2). n.a., not analyzed, n.e., not expressed, c.b.d., cannot be determined. ^∗^G2p and MOS1 co-purified with TERT fragments in other work of our group (Majerska et al., manuscript in preparation).

To obtain better insights into possible RRM cellular functions and its involvement in specific cellular processes, we screened in BY-2 protoplasts a nYFP-RRM fusion protein against a cYFP cDNA library (Lee et al., 2012). We identified one cDNA clone encoding the full length Metallothionein 2A protein (At3g09390; MT2A) and four additional cDNA clones encoding protein fragments that were in-frame with the YFP tag (**Figure 2**): (i) Modifier Of Snc1 (MOS1; At4g24680; fragment 1040–1427 aa); (ii) the putative nuclear DNA-binding protein G2p (At3g51800; 347–401 aa); (iii) Oxidation Related Zinc Finger 2 (OZF2; At4g29190; 1–68 aa); (iv) Heat Shock Cognate protein 70-1 (HSP70-1; At5g02500; 1–211 aa). In all cases, the interaction signal resembled nuclear speckles. We generated Y2H and BiFC constructs of G2p, OZF2, and HSP70-1 bearing the respective full length coding sequences to confirm interaction with the RRM protein and to test interaction with AtTERT fragments (**Figure 2B**). We were unable to obtain a full length MOS1 (1–1427 aa) construct, either by RT-PCR in our laboratory or from stock centers. Using the Y2H system we found strong interaction between OZF2 and RRM proteins, whereas MT2A, G2p, and HSP70-1 did not interact with RRM. None of the proteins interacted with any AtTERT fragments (not shown). Interestingly, using BiFC in tobacco BY-2 protoplasts we found interaction of the G2p and MT2A proteins with RRM and also with the N-terminal domain fragment RID1(1–271) of AtTERT (**Figure 2**, Supplementary Figure S2), with strong nucleolar and weak nucleoplasmic localization.

### RRM is Highly Expressed in Leaves and Reproductive Tissues

**FIGURE 3:**
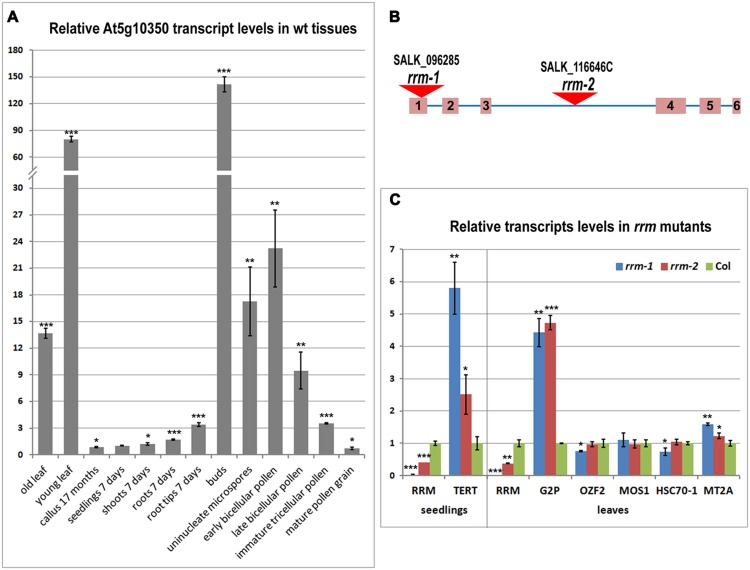
**Transcription profile of the RRM gene in tissues of wild-type plants and relative transcription of the RRM interactors in *rrm* mutants. (A)** Level of *RRM* transcripts in various wild-type (wt) plant tissues and developmental stages calculated relative to 7-days-old seedlings (Col-0) using the ΔΔCt method and ubiquitin (*ubi10*) as a reference gene. **(B)** Schematic depiction of the *RRM* gene and position of the T-DNA insertion in homozygous mutant *Arabidopsis* lines *rrm-1* and *rrm-2*. **(C)** Level of transcripts of indicated genes in 7-days-old seedlings (left panel) and 21-days-old leaves (right panel) of *rrm-1* and *rrm-2* T-DNA insertion lines calculated relative to wild-type (Col-0) using the ΔΔCt method and *ubi10* as a reference gene. Two-tailed *p*-values were calculated using the unpaired *t*-test (^∗^*p* < 0.05; ^∗∗^*p* < 0.001; ^∗∗∗^*p* = 0.0001, see Supplementary Table S2 for details).

To characterize RRM expression during plant development, we investigated the level of RRM transcripts (**Figure 3A**), including in telomerase-positive tissues. The transcripts were quantified in flower buds, calli, leaves, and 7-days-old seedlings of wild-type plants with a particular interest in detailed seedling analysis comprising whole seedlings, shoots, roots, and root tips. To quantify transcript levels in reproductive tissues, we included five pollen developmental stages (uninucleate microspores, early bicellular pollen, late bicellular pollen, immature tricellular pollen, and mature pollen). We observed RRM transcripts in all tissues tested. However, the greatest RRM transcript abundance was seen in proliferating tissues – young leaves and reproductive tissues. During pollen development, RRM transcript abundance peaked at the time of pollen mitosis I. Our RT-qPCR data confirmed previously published microarray data^15^.

### Telomere Length and Telomerase Activity in Homozygous *rrm* T-DNA Insertion Lines

To examine the role of RRM *in planta*, we analyzed *Arabidopsis* lines SALK_096285 (*rrm-1*) and SALK_116646C (*rrm-2*) harboring different T-DNA insertions in the *RRM* gene (**Figure 3B**). RT-qPCR results confirmed only *rrm-1* as a *null* allele, whereas the *rrm-2* allele caused only a partial knock-down of the *RRM* transcript (**Figure 3C**). No detectable morphological differences were observed in root length, rosette diameter, leaf number, flowering time, or silique number comparing soil-grown wild-type (Col-0) and three subsequent generations of homozygous *rrm/-* plants (not shown). Thus, RRM function does not appear to be essential for plant growth and development under these experimental conditions.

Telomere length was determined in three independent homozygous G3 mutant plants using the TRF analysis. Although telomeres in both *rrm-1/rrm-1* and *rrm-2/rrm-2* G3 generation plants were slightly longer when compared to wild-type plants (Supplementary Figure S3), a paired Student *t*-test indicated that these changes were not significant (the two-tailed *p*-values equal 0.0575 and 0.0656 for *rrm-1* and *rrm-2*, respectively). Telomerase activity in G3 generation homozygous *rrm-1* and *rrm-2* lines was tested by TRAP (telomere repeat amplification protocol) in 7-days-old seedlings. No changes in telomerase activity or processivity were observed using conventional TRAP analysis or quantitative TRAP analysis (not shown).

### Changes in Transcript Levels in Homozygous *rrm* Lines

We analyzed the transcription profiles of genes identified by our cDNA library screen in homozygous *rrm-1* and *rrm-2 Arabidopsis* mutant lines (**Figure 3C**). *G2p, MOS1, OZF2, HSP70-1*, and *MT2A* transcripts were quantified in 21-days-old leaves, a tissue with high *RRM* expression (**Figure 3C**, right panel). *AtTERT* transcripts were quantified in 7-days-old seedlings (**Figure 3C**, left panel), as there is a very low *AtTERT* transcription in *Arabidopsis* leaves (Ogrocka et al., 2012). *G2p* and *TERT* transcript levels were significantly higher in both *rrm* T-DNA insertion lines, suggesting a possible role of RRM in the regulation of these genes and/or the stability of the mRNAs encoded by these genes. *MOS1, OZF2, HSP70-1*, and *MT2A* transcripts levels were similar in mutant and wild-type plants.

Using GENEVESTIGATOR software, we identified 2102 genes putatively transcriptionally co-regulated with *RRM* and/or with at least one of its interacting partners TERT, G2P, MOS1, OZF2, HSP70-1, and MT2A, using the same conditions subset in a similar or opposite manner. A narrow subset of 137 genes showed overlapping co-regulation with at least two of these genes. We observed that *RRM* and genes encoding its presumed interactors were co-regulated with numerous ribosomal protein genes. Interestingly, most ribosomal protein genes possess a telobox, a short regulatory motif over-represented in 5′ regions of *Arabidopsis* genes with sequences identical to the repeat (AAACCCT)n of plant telomeres (Regad et al., 1994). Telobox motifs are also found in promoters of genes involved in DNA replication (Tremousaygue et al., 1999; Wang et al., 2011). Because of a possible link between the cell cycle-dependent regulation of the expression of genes encoding ribosomal proteins and telomerase (Gaspin et al., 2010), we selected for transcription analysis a subset of identified co-regulated genes in addition to genes involved in DNA replication and translation-related genes with known telobox motifs (**Table 1**). Five genes showed a 2- to 4-fold increase of transcript levels in both mutant lines, and four genes displayed an increase in the homozygous *rrm-1* line only. Interestingly, none of the genes analyzed showed significantly decreased transcript levels in the mutant lines. Transcript levels of DNA replication-related genes were not altered in either mutant line, suggesting that the telobox in the 5′ region of these genes is not a critical determinant for RRM action.

**Table 1:** Relative transcription levels of genes with identified telobox sequences and/or co-regulated with RRM interactors in homozygous *rrm* mutants.

AGI number	Gene name	*rrm-1*^∗^		*rrm-2*^∗^		Telobox	Reference	Co-regulation with RRM and its interactors (GENEVESTIGATOR score)^a^
		2^-ddCt^	*SD*	2^-ddCt^	*SD*			
**Genes encoding cytoplasmic ribosomal proteins**								
At1g23290	RPL27a	**2.08**	0.37	**2.56**	1.23	Yes	In this work	RRM (0,73), G2P (0,89), HSP70-1 (0,78)
At1g72370	RP40	**4.72**	0.13	**3.42**	0.22	Yes	Tremousaygue et al., 1999; Manevski et al., 2000	RRM (0,75), G2P (0,92), HSP70-1 (0,77)
At3g04840	RPS3Ae family	1.78	0.10	1.34	0.11	Yes	In this work	RRM (0,78), G2P (0,89), HSP70-1 (0,76)
At3g25520	RPL5	1.66	0.07	1.32	0.11	Yes	In this work	RRM (0,84), G2P (0,90), HSP70-1 (0,84)
At5g39740	RPL5b	1.41	0.01	1.12	0.13	Yes	In this work	RRM (0,87), G2P (0,90), HSP70-1 (0,75)
At3g47370	RPS10p/S20e family	**2.30**	0.01	1.45	0.10	Yes	In this work	RRM (0,73), MOS1 (-0,81), G2P (0,90), HSP70-1 (0,73)
At3g49010	BBC1	**3.70**	0.55	1.86	0.24	Yes	In this work	RRM (0,83), G2P (0,92), HSP70-1 (0,77)
At3g51190	RPL2 family	**2.41**	0.2	1.89	0.11	Yes	In this work	n.a.
At3g56340	RPS26e family	1.38	0.03	1.17	0.08	Yes	In this work	RRM (0,75), HSP70-1 (0,68)
At3g60770	RPS13/S15 family	1.95	0.12	1.44	0.07	Yes	In this work	RRM (0,78), G2P (0,90), HSP70-1 (0,80)
At4g00810	RPS60 family	**2.39**	0.09	**2.25**	0.10	Yes	Tremousaygue et al., 1999	MOS1 (-0,88)
At4g09800	RPS18C	**2.69**	0.23	**2.35**	0.05	Yes	Tremousaygue et al., 1999	No co-regulation
**Genes encoding plastid ribosomal proteins**								
At1g79850	PRPS17	0.68	0.02	1.68	0.28	Yes	Tremousaygue et al., 1999	No co-regulation
At2g33450	PRPL28	**2.62**	0.55	1.33	0.15	Yes	Tremousaygue et al., 1999	No co-regulation
**Genes encoding translation factors**								
At1g07940	EF1A family	1.88	0.01	0.87	0.01	Yes	Tremousaygue et al., 1999	No co-regulation
At1g54290	TIF SUI1 family	**2.77**	0.23	**2.43**	0.34	Yes	Tremousaygue et al., 1999	No co-regulation
**DNA replication-related genes**								
At1g07270	CDC6b	0.98	0.11	1.03	0.08	No	In this work	No co-regulation
At1g07370	PCNA1	1.13	0.07	1.13	0.11	Yes	Manevski et al., 2000	No co-regulation
At1g44900	MCM2	0.97	0.13	0.99	0.12	Yes	In this work	No co-regulation
At5g46280	MCM3	0.68	0.02	0.95	0.08	Yes	In this work	No co-regulation
At2g16440	MCM4	0.94	0.01	0.94	0.06	No	In this work	No co-regulation
At2g07690	MCM5	0.95	0.07	0.99	0.08	No	In this work	No co-regulation
At5g44635	MCM6	0.72	0.03	0.90	0.12	Yes	In this work	n.a.
At4g02060	MCM7	1.03	0.07	0.97	0.13	Yes	In this work	No co-regulation
**Other co-regulated genes**								
At2g19480	NAP1;2	1.85	0.15	1.42	0.15	Yes	In this work	RRM (0,79), HSP70-1 (0,75)
At3g54230	SUA	1.54	0.22	1.25	0.09	Yes	In this work	TERT (0,88), MOS1 (0,95)
At4g17520	Hyaluronan family	0.60	0.21	1.55	0.13	Yes	In this work	RRM (0,77), G2P (0,93)
At5g14790	ARM superfamily	0.76	0.05	0.87	0.08	No	In this work	TERT (-0,84)

*^a^n.a., data not available; ^∗^more than two-fold change in transcript level (2^-ddCt^) is highlighted; *SD*, standard deviation.*

## Discussion

The identification of an RRM protein as a nuclear interactor with the CTE domain of AtTERT in tobacco BY2 protoplasts (Lee et al., 2012) was somehow surprising. However, yeast genome-wide screens (Askree et al., 2004; Gatbonton et al., 2006; Ungar et al., 2009) revealed a number of proteins that influenced telomere length and which were involved in numerous cellular processes without a known link to telomere maintenance. Among these were human proteins involved in RNA metabolism and transcription pathways connected with non-telomeric functions of telomerase (see Majerska et al., 2011 for review). A conserved protein structure comprising the coiled-coil N-terminus, a single internal RRM domain, and the C-terminal region with a nuclear localization signal classified the RRM protein as a nuclear poly(A) binding protein (PABPN, Eliseeva et al., 2013). Recently, a RRM protein was identified as an interactor with AtCSP3 (COLD SHOCK DOMAIN PROTEIN 3; Kim et al., 2013), and hnRNP-like proteins (Arabidopsis Interactome Mapping Consortium, et al., 2011). Here, we verified RRM interaction with the CTE domain of AtTERT using BiFC in *Arabidopsis* protoplasts, but their direct interaction was not observed in yeast. Known technical differences of both screening systems suggest that the *in vivo* interaction is mediated by an additional protein absent in the yeast cell, or is facilitated by post-translational modification missing in yeast. Analysis of T-DNA insertion mutant lines showed no obvious changes in telomere lengths and telomerase activity, suggesting that the RRM protein is not essential for telomere maintenance. The observed interaction with telomerase may reflect possible non-telomeric functions.

The localization of the RRM-YFP signal in nuclear speckles and the observation that all described BiFC RRM interactions (and interaction with CSP3 protein, Kim et al., 2013) are nuclear-localized suggest that RRM is in fact a PABPN. Human PABPN1 localizes to nuclear speckles as a consequence of RNA poly(A) binding (Calado and Carmo-Fonseca, 2000). Our observations indicate a conserved structure-localization relationship of PABPNs across eukaryotic species. Nucleic acid binding of RRM-containing proteins is often mediated by a pair of RRM domains (Deo et al., 1999; Kwon and Chung, 2004). On the other hand, *Xenopus laevis* XlePABP2 and *Citrus sinensis* CsPABPN1, PABPNs that share similar structure with RRM protein, both bind poly(A) as monomers and undergo a dimer-monomer transition upon poly(A) binding (Song et al., 2008; Domingues et al., 2015). We visualized RRM protein dimerization in tobacco BY-2 protoplasts and observed the same nuclear speckle pattern as with RRM-YFP localization. Moreover, we demonstrated that RRM protein dimerizes through its C-terminal region. This last observation contradicts the published dimerization model of hPABPN1 (Ge et al., 2008), which identified the amino acid residues responsible for self-interaction within the RRM domain.

We revealed a possible connection between RRM and non-canonical telomerase functions by identifying interaction partners of RRM. Screening for nYFP-RRM protein–protein interactions against a cYFP-tagged cDNA library identified five putative RRM interactors with various annotated functions such as transcription regulation (OZF2), epigenetic regulation (MOS1), mRNA catabolism (MOS1), RNA methylation (G2p), protein nuclear import (G2p), protein folding (HSP70-1), proteolysis (G2p), cellular copper ion homeostasis (MT2A), or metabolism (MOS1, HSP70-1). In all five cases, the interaction localization pattern resembles nuclear speckles, as observed for RRM-YFP subcellular localization. A G2p-GFP fusion protein was previously localized in the nucleus (Zhang et al., 2005). However, the subcellular localization of other RRM interactors has not previously been described. Interestingly, other data from our group showed that G2p and MOS1 co-purify with TERT (**Figure 2**, Majerska et al., manuscript in preparation) and G2p and MT2A interact with TERT(RID1) using BiFC in tobacco BY-2 protoplasts (**Figure 2B**, Supplementary Figure S2), suggesting co-existence of TERT, RRM, G2p, MOS1, and MT2A in a multiprotein complex.

Analysis of telomere length and telomerase activity in homozygous *rrm-1* and *rrm-2* T-DNA insertion mutants indicated that the RRM protein was not important for the canonical telomeric functions of telomerase. On the other hand, *TERT* transcripts were elevated in homozygous *rrm* mutants, and TERT and RRM may share binding partners such as G2p, MOS1, and MT2A. These observations suggest that RRM plays a role in non-telomeric activities of telomerase. Interestingly, the G7 generation of a homozygous *tert* T-DNA insertion line showed increased *OZF2* and *MT2A* transcript levels (Amiard et al., 2014). PABPNs are implicated in processes that might be crucial for post-transcriptional regulation of gene expression. Our qPCR analyses indicated that RRM might generally function as a negative regulator of gene expression, because none of the 34 genes analyzed here showed a significant decrease in transcript levels in homozygous *rrm* mutants. Increased levels of *TERT* and *G2p* transcripts in homozygous *rrm* mutant lines indicated a possible feedback mechanism in RRM-TERT and RRM-G2p interactions. Moreover, nine ribosomal and translation-related genes also showed significantly increased transcript levels in a *rrm* mutant background. We have further analyzed these nine genes for transcript level perturbations across different conditions using GENEVESTIGATOR. *RPL2* transcripts were stable across various conditions. Interestingly, the transcript levels of the other eight genes changed more than two-fold in response to salt stress in the *myb44* T-DNA insertion line (Jung et al., 2008). RP40, RPL27A, RPS10p/S20e, BBC1, and RPL2 form a protein interaction network (STRING database^16^, Szklarczyk et al., 2015) and are mutually co-regulated. The RRM interactome, subcellular localization, and co-regulation profile showing that the expression of the majority of its co-regulated genes contain telobox motifs in their promoters, further support the hypothesis that RRM may function in mediating non-telomeric (non-canonical) functions of telomerase. DNA replication-related genes were not co-regulated with genes encoding RRM or its interactors, and they also did not show changes in transcript abundance in a homozygous *rrm* background. These results suggest that the telobox in promoters of these genes are not a critical determinant of RRM action. Regulation of translation-related genes is generally important for the regulation of protein synthesis and consequently for cell growth. These genes regulate tumor onset and progression (reviewed in Loreni et al., 2014), further indicating a possible link between RRM and its interactors to TERT non-telomeric functions. Our results support a functional connection between RRM and its interaction partners in plant regulatory protein complex(es).

## Conclusion

The RRM protein was previously identified as an interaction partner of AtTERT. However, telomere length shortening in RRM protein knockout mutant plants was not significant. By screening a cDNA library using cYFP-RRM as a bait, we identified five interaction partners; two of them interacted also with TERT fragments. Investigation of the subcellular localization and protein structure suggested that RRM-protein may function as a nuclear poly(A)-binding protein. Transcriptional profiling revealed a possible involvement of RRM-protein in the regulation of a subset of ribosomal and translation-related genes. Most of these genes contain a telobox motif in their promoters. *G2p* and *TERT* transcript levels were significantly higher in *rrm*/- knockout mutants, suggesting a possible role for RRM in the regulation of these genes and/or the stability of the mRNAs encoded by these genes. Overlaps of the RRM and TERT interactome, subcellular localization of protein–protein interactions, and co-regulation profiles support the hypothesis that RRM may be involved in mediating non-canonical telomerase functions.

## Author Contribution

LD performed most of experiments except pollen RT-qPCR performed by DH and RR. L-YL was involved in cDNA screening, ES was involved in cloning. ES and SBG designed the study.

## Conflict of Interest Statement

The authors declare that the research was conducted in the absence of any commercial or financial relationships that could be construed as a potential conflict of interest.

## Footnotes

1 https://www.duchefa-biochemie.com/

2 http://www.thermoscientificbio.com/finnzymes

3 www.kapabiosystems.com

4 http://www.lifetechnologies.com

5 http://www.arabidopsis.org/

6 www.clontech.com

7 www.sigmaaldrich.com

8 www.roche.com

9 www.qiagen.com

10 www.thermofisher.com

11 www.appliedbiosystems.com

12 https://genevestigator.com/gv/

13 http://www.ncbi.nlm.nih.gov/

14 http://www.string-db.org/

15 http://bbc.botany.utoronto.ca/

16 http://www.string-db.org/
