# Supplementary material for: cDNA Library Screening Identifies Protein Interactors Potentially Involved in Non-Telomeric Roles of Arabidopsis Telomerase
Source: Front Plant Sci. 2015 Nov 12;6:985. doi: 10.3389/fpls.2015.00985 (PMC4641898; doi:10.3389/fpls.2015.00985)
Supplement: Supplementary file 3 [file Image_1.PDF]

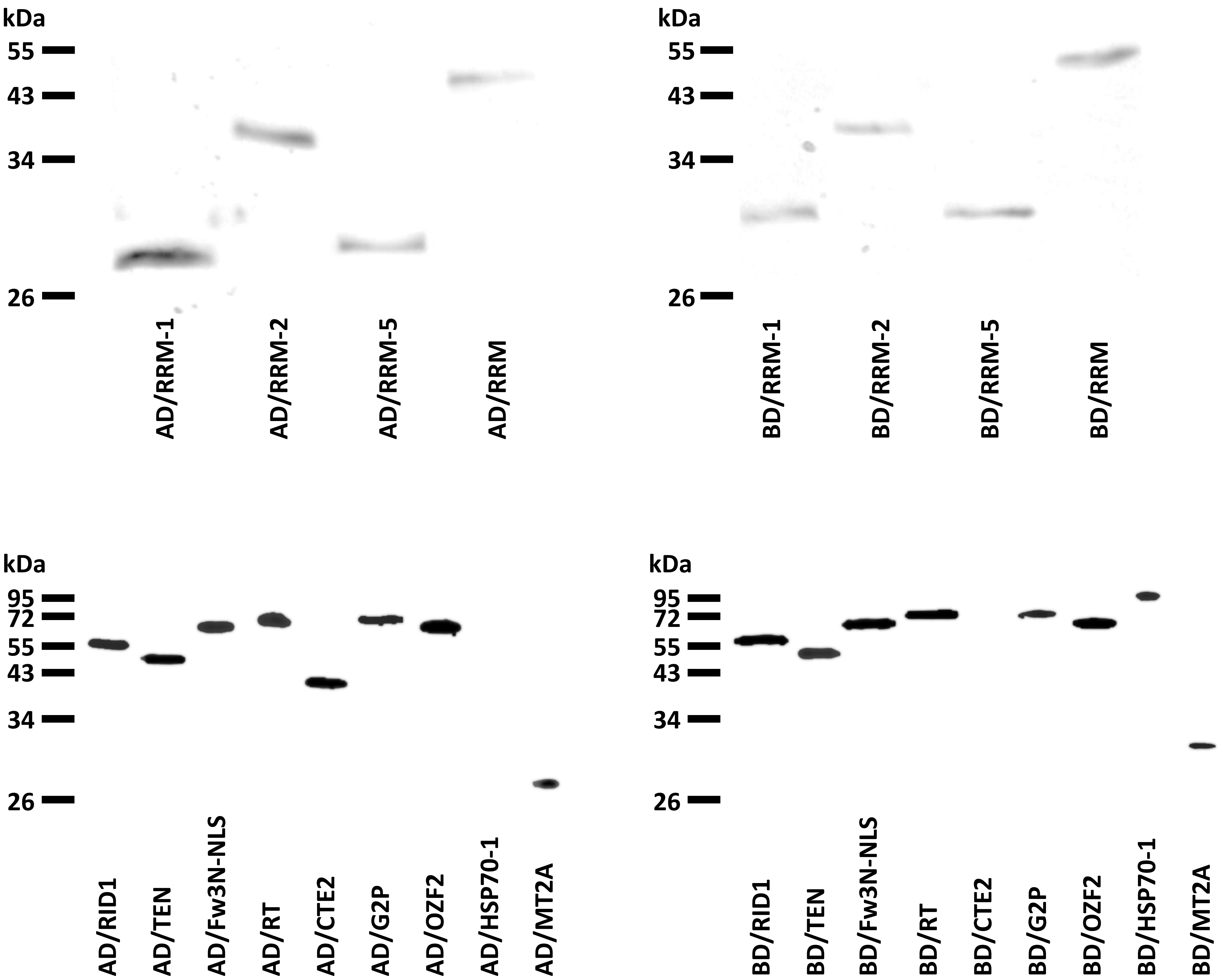

Supplementary Figure S1. Expression of proteins tested for interactions in yeast. Constructs encoding full-length RRM protein, RRM-1, -2, -5 protein fragments, TERT fragments (RID1, TEN, Fw3N-NLS, RT, CTE2, Zachova *et al.,* 2013), G2P, OZF2, HSP70-1, and MT2A proteins fused with the GAL4 activation domain (AD, left panels) or the GAL4 DNA-binding domain (BD, right panels) are expressed in yeast cells. AD- and BD-fusion proteins were detected by immunoblotting using mouse anti-HA and mouse anti-myc antibodies binding to the epitope tags of these proteins, respectively. Note the AD/HSP70-1 and BD/CTE2 constructs were not expressed in yeast.
