# Supplementary material for: cDNA Library Screening Identifies Protein Interactors Potentially Involved in Non-Telomeric Roles of Arabidopsis Telomerase
Source: Front Plant Sci. 2015 Nov 12;6:985. doi: 10.3389/fpls.2015.00985 (PMC4641898; doi:10.3389/fpls.2015.00985)
Supplement: Supplementary file 4 [file Image_2.PDF]

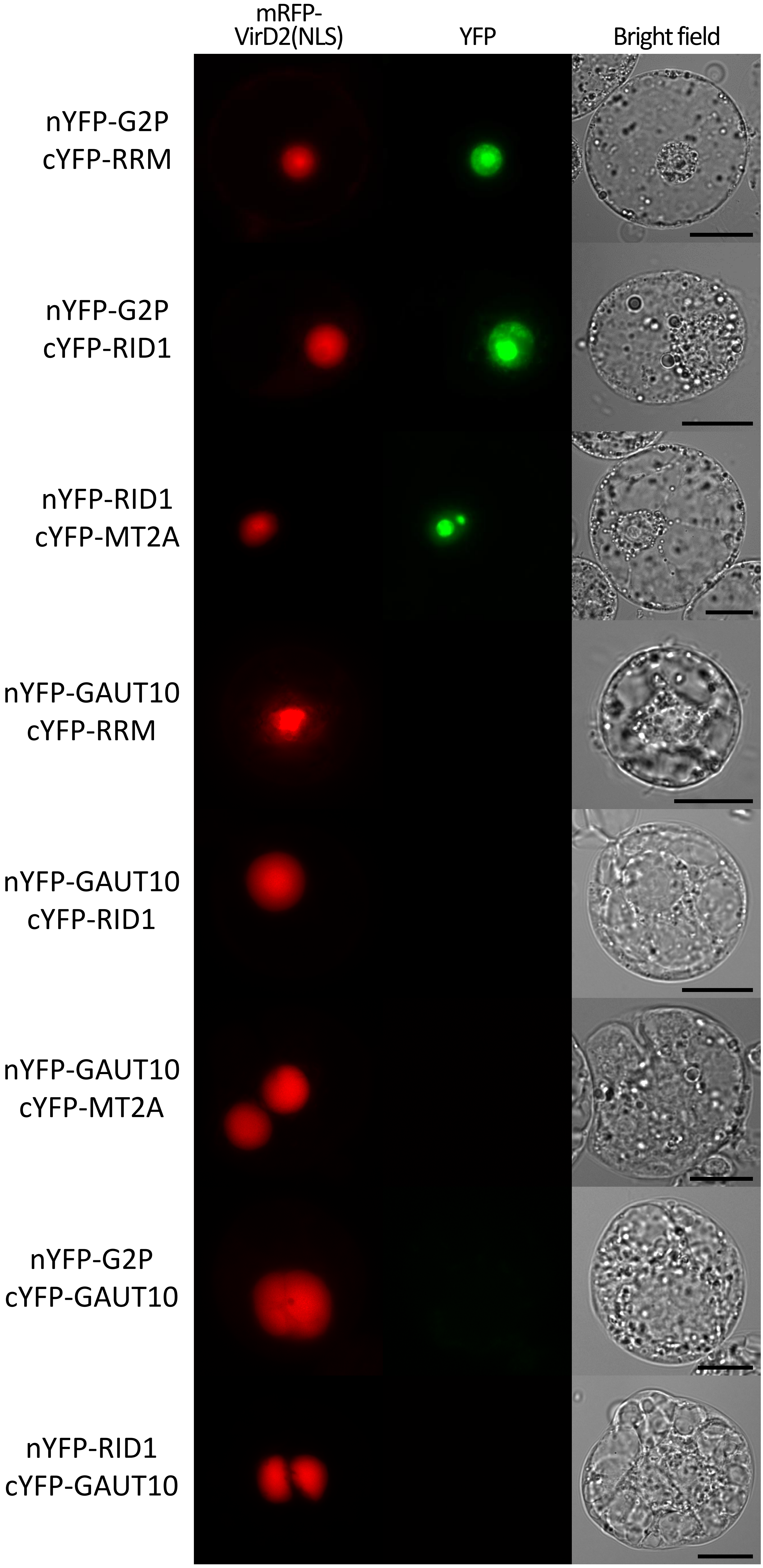

Supplementary Figure S2. BiFC interactions of nYFP-G2p with cYFP-RRM, and cYFP-TERT(RID1) or cYFP-MT2A with nYFP-TERT(RID1). Tobacco BY-2 protoplasts were co-transfected with a nYFP construct, a cYFP construct, and a mRFP-VirD2(NLS) nuclear marker. nYFP- and cYFP-GAUT10 constructs served as negative controls. YFP fluorescence (green), mRFP fluorescence (red). Scale bars indicate 20 µm.
