# Supplementary material for: cDNA Library Screening Identifies Protein Interactors Potentially Involved in Non-Telomeric Roles of Arabidopsis Telomerase
Source: Front Plant Sci. 2015 Nov 12;6:985. doi: 10.3389/fpls.2015.00985 (PMC4641898; doi:10.3389/fpls.2015.00985)
Supplement: Supplementary file 5 [file Image_3.PDF]

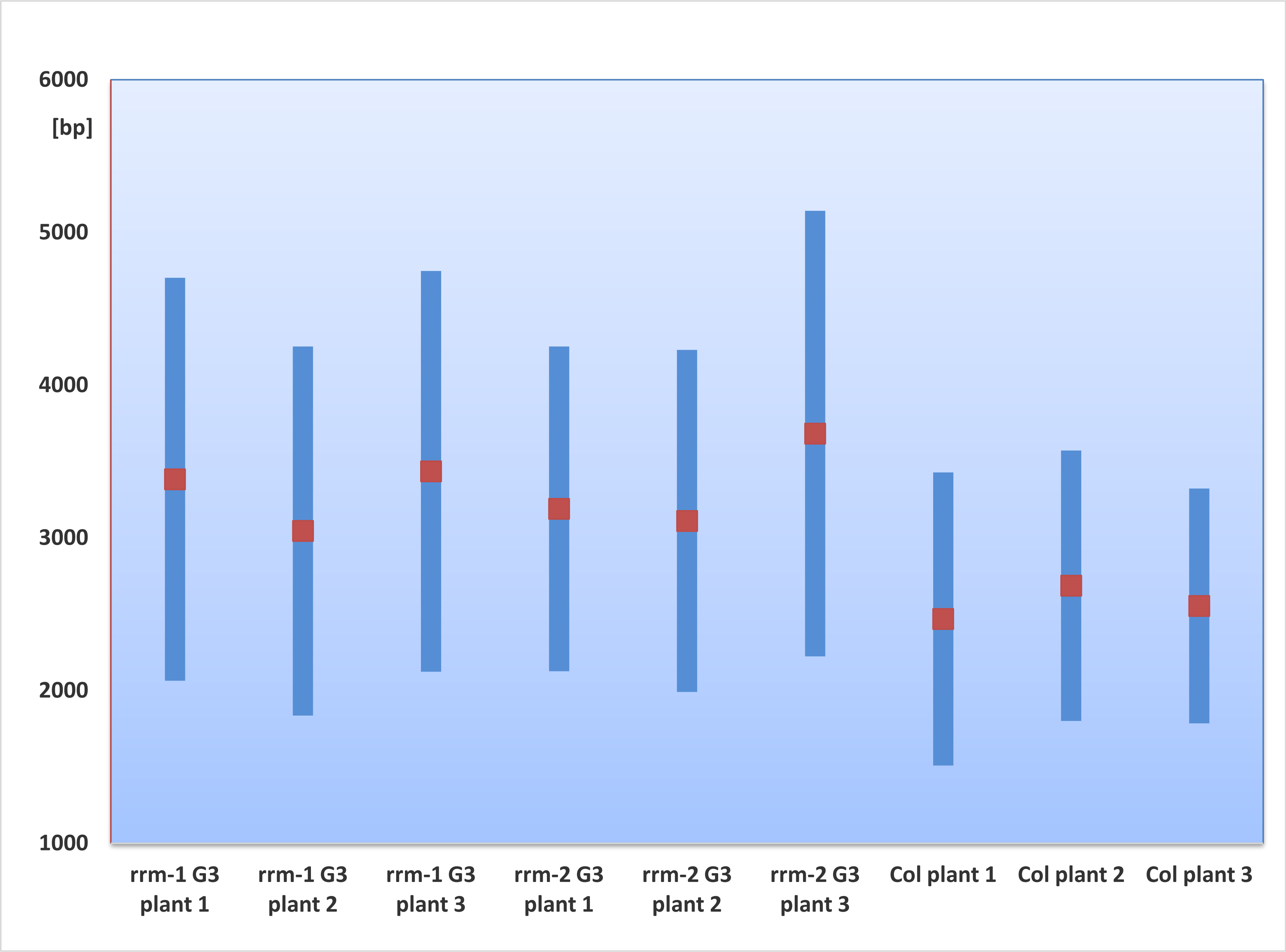

Supplementary Figure S3. Telomere length in *rrm-1* and *rrm-2* T-DNA insertion lines determined by terminal restriction fragment (TRF) Southern blots analysis followed by analysis using the TeloTool software. Although telomeres in both *rrm-1* and *rrm-2* G3 plants are slightly longer when compared to those of wild-type Col-0 plants, a paired Student t-test evaluated these changes in telomere lengths as not significant (the two-tailed P value equals 0.0575 and 0.0656 for *rrm-1* and *rrm-2*, respectively). TRF signal – blue rectangle, mean value – red square.
